# Novel functional proteins coded by the human genome discovered in metastases of melanoma patients

**DOI:** 10.1007/s10565-019-09494-4

**Published:** 2019-10-10

**Authors:** Aniel Sanchez, Magdalena Kuras, Jimmy Rodriguez Murillo, Indira Pla, Krzysztof Pawlowski, A. Marcell Szasz, Jeovanis Gil, Fábio C. S. Nogueira, Yasset Perez-Riverol, Jonatan Eriksson, Roger Appelqvist, Tasso Miliotis, Yonghyo Kim, Bo Baldetorp, Christian Ingvar, Håkan Olsson, Lotta Lundgren, Henrik Ekedahl, Peter Horvatovich, Yutaka Sugihara, Charlotte Welinder, Elisabet Wieslander, Ho Jeong Kwon, Gilberto B. Domont, Johan Malm, Melinda Rezeli, Lazaro Hiram Betancourt, György Marko-Varga

**Affiliations:** 1 Section for Clinical Chemistry, Department of Translational Medicine, Skåne University Hospital Malmö, Lund University, 205 02 Malmö, Sweden; 2 Clinical Protein Science & Imaging, Biomedical Centre, Department of Biomedical Engineering, Lund University, BMC D13, 221 84 Lund, Sweden; 3 Biology, Warsaw University of Life Sciences, Warsaw, Poland; 4 Cancer Center, Semmelweis University, Budapest, 1083 Hungary; 5 Proteomics Unit, Department of Biochemistry, Federal University of Rio de Janeiro, Rio de Janeiro, Brazil; 6 Laboratory of Proteomics, LADETEC, Institute of Chemistry, Federal University of Rio de Janeiro, Rio de Janeiro, Brazil; 7 European Molecular Biology Laboratory, European Bioinformatics Institute (EMBL-EBI), Wellcome Trust Genome Campus, CB10 1SD Hinxton, Cambridge, UK; 8 AstraZeneca R&D, Mölndal, Sweden; 9 Division of Oncology and Pathology, Department of Clinical Sciences Lund, Lund University, 221 85 Lund, Sweden; 10 Department of Surgery, Clinical Sciences, Skåne University Hospital, Lund University, Lund, Sweden; 11 Department of Hematology, Oncology and Radiation Physics, Skåne University Hospital, Lund, Sweden; 12 Department of Analytical Biochemistry, Faculty of Science and Engineering, University of Groningen, Groningen, The Netherlands; 13 Department of Biotechnology, Yonsei University, Seoul, South Korea

**Keywords:** Melanoma, Missing proteins, Tissue, Biobank, Proteomics, Mass spectrometry

## Abstract

In the advanced stages, malignant melanoma (MM) has a very poor prognosis. Due to tremendous efforts in cancer research over the last 10 years, and the introduction of novel therapies such as targeted therapies and immunomodulators, the rather dark horizon of the median survival has dramatically changed from under 1 year to several years. With the advent of proteomics, deep-mining studies can reach low-abundant expression levels. The complexity of the proteome, however, still surpasses the dynamic range capabilities of current analytical techniques. Consequently, many predicted protein products with potential biological functions have not yet been verified in experimental proteomic data. This category of ‘missing proteins’ (MP) is comprised of all proteins that have been predicted but are currently unverified. As part of the initiative launched in 2016 in the USA, the European Cancer Moonshot Center has performed numerous deep proteomics analyses on samples from MM patients. In this study, nine MPs were clearly identified by mass spectrometry in MM metastases. Some MPs significantly correlated with proteins that possess identical PFAM structural domains; and other MPs were significantly associated with cancer-related proteins. This is the first study to our knowledge, where unknown and novel proteins have been annotated in metastatic melanoma tumour tissue.

## Introduction

Metastatic melanoma is an aggressive disease; previously known to resist most types of therapies. However, the development of targeted therapies in tumours with BRAF mutations has revolutionised treatment. Nevertheless, a significant number of patients with BRAF V600 metastatic melanoma experience relapse within a few months after treatment with the combination of BRAF and MEK inhibitors (Pascale et al. 2018). With the advent of immunotherapy, a significant improvement in survival has become evident (Eroglu et al. 2018). Nonetheless, the disease often overcomes therapeutic blockage of the immune system.

New and promising classification systems and methods have emerged that have enabled stratification of patients into refined prognostic clusters. Such approaches undoubtedly complement available therapies. As such, a more uniform prognosis is provided and, more importantly, an improved response to treatment (Pimiento et al. 2013; Tímár et al. 2016; Dimitriou et al. 2018). Based on genetic analyses, cutaneous melanomas are divided into four classes: BRAF-mutated, RAS-mutated, NF-1-mutated tumours, and triple wild-type (Cancer Genome Atlas Network et al. 2015). Independent of these sub-groups, immune therapy with check-point inhibitors across tumours has resulted in an improved outcome. Applying transcriptomic profiling and using paired-end massively parallel sequencing of cDNA together with analyses of high-resolution chromosomal copy number data, 11 novel melanoma gene fusion products and 12 novel readthrough transcripts have been identified. From this RNA-seq analysis, a surprisingly high mutational burden was described in melanoma that was crucial for tumour progression (Berger et al. 2010).

Heterogeneity, clonal expansion and evolutionary processes are further key phenomena that may be responsible for the resistance mechanism of cancer (Marcell Szasz et al. 2019; Turajlic et al. 2019; Swanton 2018). A deeper understanding of single individual tumour can reveal important pieces of the entire puzzle. For example, immunotherapies are now administered in earlier stages and it was shown that neoadjuvant ipilimumab + nivolumab expand more tumour-resident T cell clones than adjuvant application (Blank et al. 2018). The adverse effects have prompted further studies and approaches to apply immunotherapies in a safer manner (Bosman et al. 2010).

In order to address unsolved clinical drawbacks, alternative research approaches have emerged. Proteomics has been successfully applied to several biological scenarios as an integral part of multi-omics studies in system biology and medicine (Collins and Varmus 2015; Chen and Snyder 2013).

By nature, proteins are highly complex. Therefore, as a consequence of the dynamic range and sensitivity limits of current proteomic techniques, many predicted protein products have not yet been identified in proteomic experiments. These proteins could provide essential clues to aid interpretation of biological processes and potentially drive new avenues of research and therapeutic strategies to solve remaining clinical problems.

In 2016, the Chromosome-centric Human Proteome Project (C-HPP) launched an initiative to accelerate the identification and assignment of these ‘missing proteins’(MPs) (Omenn et al. 2017). The proteins were divided into five groups according to the level of protein existence (PE). PE1 contains proteins identified by mass spectrometry, 3D structure, immunohistochemistry, and/or amino acid sequencing. PE2 refers to transcript expression, but not protein expression. Proteins annotated in PE3 do not have any protein or transcript evidence in humans; however, there are similar sequences that have been reported in other species. PE4 proteins are hypothesised from gene models, and the PE5 group contains predicted protein sequences with uncertain evidence and is mostly associated with pseudogenes (Paik et al. 2018).

**Fig. 1:**
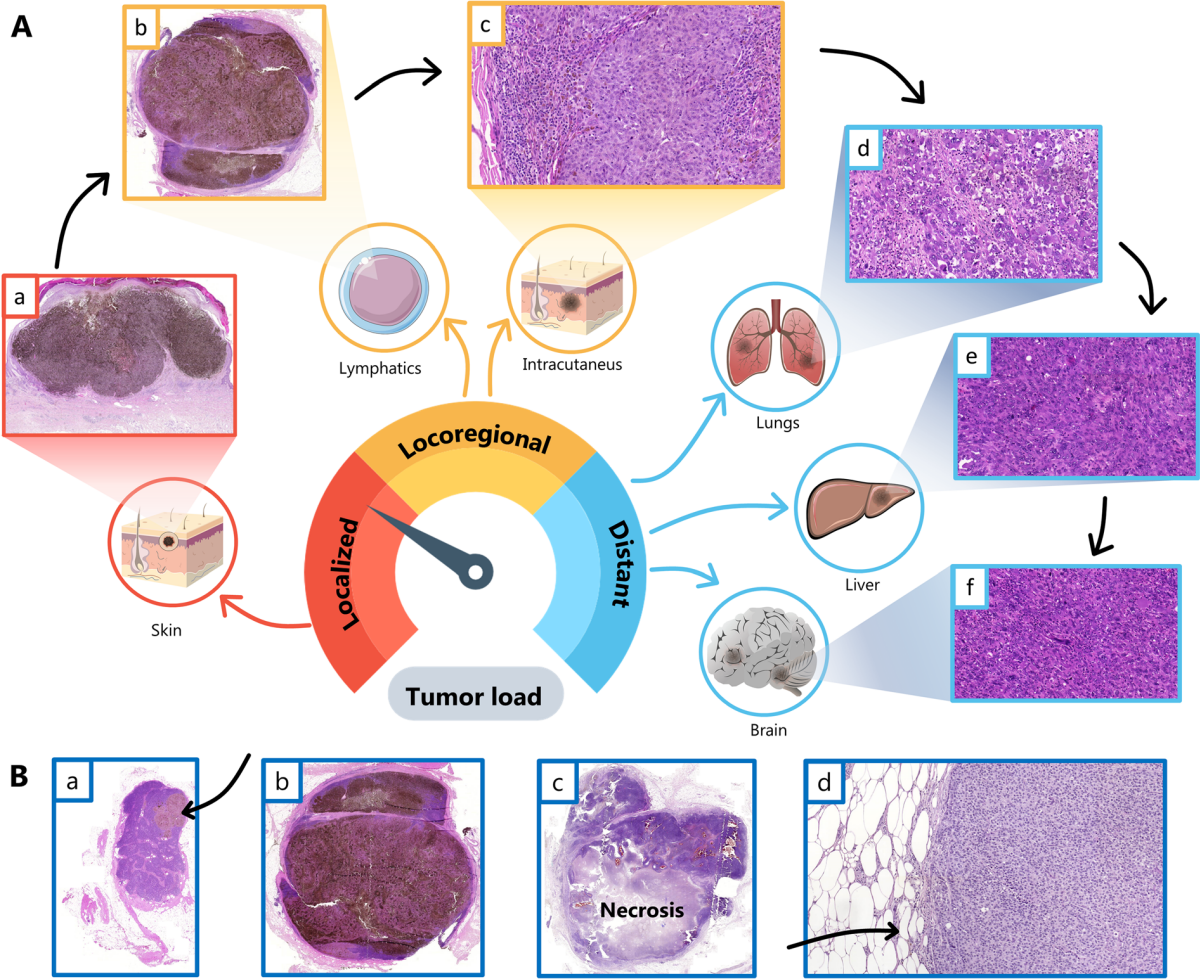
(A) Life history of a melanoma. The image depicts the evolving progression of a malignant melanoma originating from the skin, spreading to the lymphatic system and giving rise to transit (intracutaneous) and distant metastases (lung, liver, and eventually brain). The histological images in chronological order: (a) primary nodular melanoma (1×, HE), (b) lymph node metastasis (1×, HE), (c) lymph node metastasis composed of epithelioid tumour cells (20×, HE), (d) lung metastasis in fibrotic background and presence of tumour infiltrating lymphocytes (20×, HE), (e) liver metastasis—note the brisk mitotic activity and morphological change in cell shape, spindly melanoma cells (20×, HE), (f) brain metastasis of spindle and ‘monster’ melanocytes (20×, HE). (B) Metastatic melanoma in the lymphatic system in four patients. (a) Small and circumscribed melanoma in a lymph node (1×, HE). (b) Large pigmented melanoma filling the lymph node (1×, HE). (c) Large melanoma with necrotic areas (1×, HE). (d) Melanoma breaking the capsule of the lymph node and infiltrating the neighbouring tissue (20×, HE)

The samples in this study are a part of the BioMel biobank, governed by Lund Melanoma Study Group (LMSG). It is a collection of blood and tissue (primary and metastases) samples with detailed clinical information from patients diagnosed with malignant melanoma in Southern Sweden. Since 2013, the sample collection is prospective, including fresh frozen tissue and blood. Our biobank coupled high-end proteomic platform was used to study the melanoma tumour tissues (Welinder et al. 2015, 2017; Welinder et al., 2014a, b; Gil et al. 2019; Kuras et al. 2018; Murillo et al. 2018). We used histopathological characterisation and a genomic data–directed proteomic strategy to successfully identify a protein expression pattern that was associated with improved survival prognostics in lymph node samples from stage 3 malignant melanoma patients (Betancourt et al. 2019). A progression from locoregional to distantly spread disease was witnessed throughout the years (Fig. 1). In a more recent work, a deeper investigation has been undertaken to identify proteins in metastatic disease, namely, those that may be responsible for further progression (Gil et al. 2019).

As a consequence of the high diversity of individuals, it is crucial to perform large-scale analyses of clinical samples. This enables the identification of the highest number of proteins possible, including proteins that have never been previously reported by mass spectrometry.

In the current study, a novel data set of 33 proteins is presented. These proteins were identified across 140 lymph node metastatic tumour samples from malignant melanoma patients. All identified proteins are currently annotated in Nextprot (Gaudet et al. 2015) as ‘missing proteins’. According to the HUPO guidelines, 9 of the proteins were confidently identified by mass spectrometry. Association clusters were constructed to pinpoint predicted functional annotations for these proteins.

## Materials and methods

This study was approved by the Regional Ethical Committee at Lund University, Southern Sweden, approval numbers: DNR 191/2007, 101/2013 and 2015/266, 2015/618. All patients involved in the study provided written informed consent. The malignant melanoma lymph node metastases were collected from patients undergoing surgical resection at Lund University Hospital, Sweden. Out of the 140 tumours included in this study, only four received any of the novel therapies. Nevertheless, the majority of the patients enrolled in the study died due to the progression of the disease. Histopathological analysis of the tissues was performed by a board-certified pathologist (Gil et al. 2019). Protein extraction and digestion were performed according to the protocol described by Kuras et al. (2018), and the resultant peptides were labelled with TMT 11-plex reagents (Thermo Fisher Scientific, San Jose, CA, USA) according to the instructions provided. Labelled peptides were separated into 24 fractions by basic reversed-phase liquid chromatography on a Phenomenex Aeris C8 column (100 mm × 2.1 mm, 3.6-μm particles) using an Agilent 1100 HPLC system.

LC-MS/MS analysis was performed on an UltiMate 3000 RSLCnano system coupled to a Q Exactive HF-X mass spectrometer (Thermo Fisher Scientific, San José, CA, USA). Data were acquired in DDA, with the ADP set to off, selecting the top 20 precursors. Full MS scans were acquired over *m*/*z* 350–1400 range at a resolution of 120,000 (at *m*/*z* 200), target AGC value of 3 × 10^6^, maximum injection time of 50 ms, and normalised collision energy of 34%. The tandem mass spectra were acquired in the Orbitrap mass analyser with a resolution of 45,000, a target ACG value of 1 × 10^3^ and a maximum injection time of 86 ms. An isolation window of 0.7 *m*/*z* was used and fixed first mass was set to110 *m*/*z*. Data were processed with Proteome Discoverer v2.3 (Thermo Fisher Scientific, San José, CA, USA) and searched against the *Homo sapiens* UniProt revised database (2018-10-01), including isoforms, with Sequest HT. Cysteine carbamidomethylation was set as fixed modification and methionine oxidation, protein N-terminal acetylation, TMT6plex (+ 229.163 Da) at N-terminal and lysine residues were set as dynamic modifications. Peptide mass tolerance for the precursor ions and MS/MS spectra were 10 ppm and 0.02 Da, respectively.

Protein evidence (PE) was determined using the criteria adopted from neXtProt and the Chromosome-centric Human Protein Project (C-HPP) (Omenn et al. 2018).

### Bioinformatics

#### Missing protein identification

**Fig. 2:**
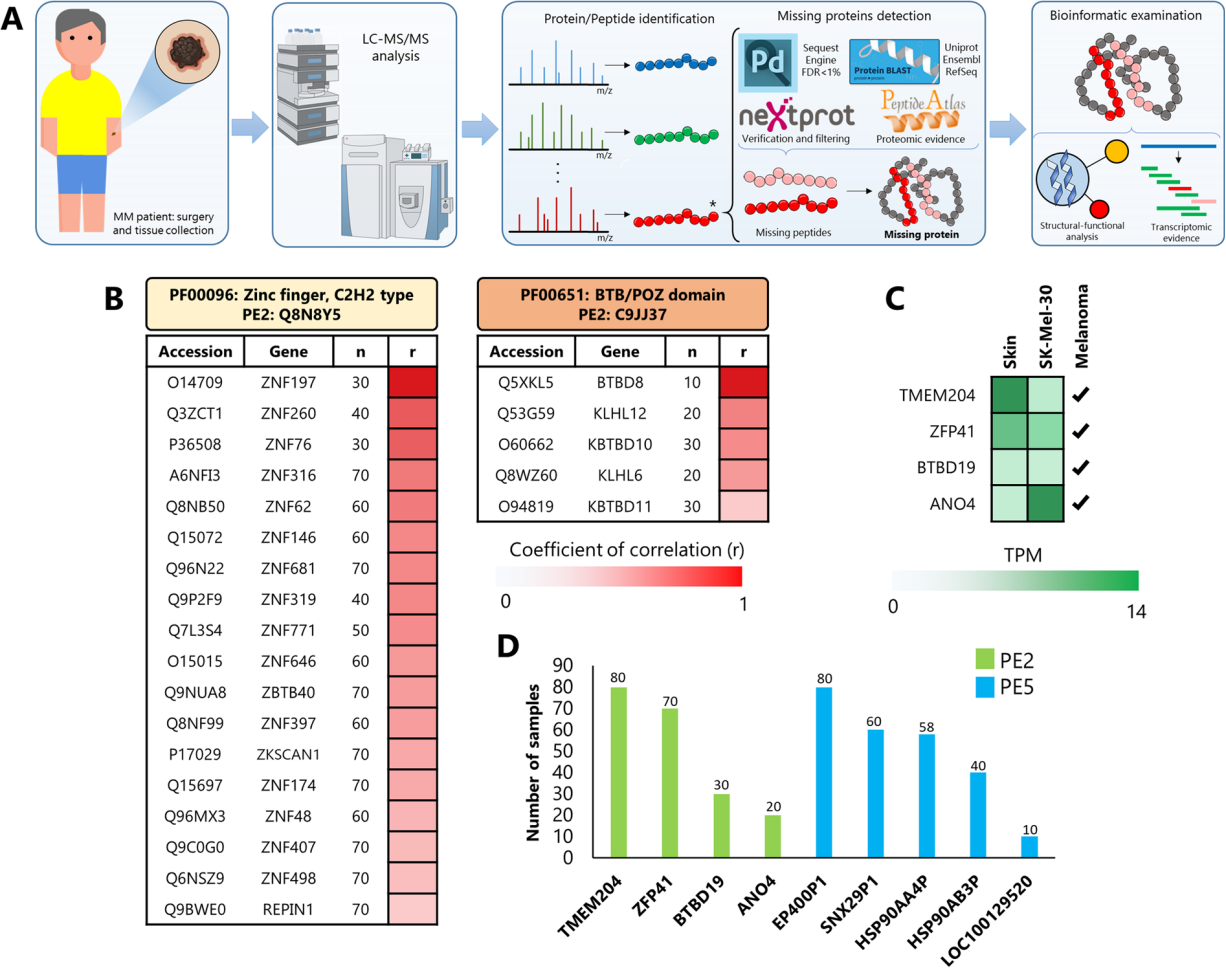
Experimental workflow and information related to the nine ‘missing proteins’ reported. **(**A) A total of 140 MM tissues were analysed by LC-MS/MS. MS/MS spectra were contrasted with available databases and with annotation levels of protein identification (PE1-5). Missing proteins were evaluated in terms of peptide length, number of peptides, structural and functional analysis, and transcriptomic evidence comparison. (B) Protein spearman correlation based on the expression of two of the PE2 proteins and proteins belong to the Zing Finger, H2H2 type, and BTP/POZ domain. The number of samples and the *r* value for the spearman correlation are represented by *n* and *r* respectively. (C) Evidence-based on The Human Atlas for the four PE2 missing proteins in skin tissues, melanoma cell lines (SK-Mel-30), or melanoma tissues, TPM (Transcripts per Kilobase Million). (D) Frequency of identification across the 140 tumour samples; *Y*-axis represents the number of samples where the proteins were identified

Peptide-spectrum match (PSM), peptide, and protein identifications were filtered to less than 1% FDR. Identification and sorting of unique peptides were carried using the neXtProt tool ‘Peptide uniqueness checker’ (https://www.nextprot.org/tools/peptide-uniqueness-checker) for all peptide sequences from proteins classified by neXtProt as P2-P5. PSMs mapping to missing proteins were also manually inspected. All novel peptides (peptides without MS evidence) were aligned using BLASTp (version: 2.7.1) to three different databases UniProt (release date: 2018), Ensembl (release date: 2019), and RefSeq (release date: 2019) as previously suggested (Nesvizhskii 2014). All possible peptide variants were filtered using the following filters: identity score higher 70, less than 2 amino acids substitutions with respect to the original novel peptide, and theoretical mass within 10 ppm compared with the precursor mass. In addition, novel unique peptides were searched in PeptideAtlas (http://www.peptideatlas.org) to explore previously reported evidence in public proteomics data (Fig. 2a).

#### Structural and functional identification

Structural domains in the novel proteins were identified by the conserved domain search tool (Marchler-Bauer et al. 2017). Additionally, structural domains were predicted by the FFAS and HHpred algorithms (Jaroszewski et al. 2011; Zimmermann et al. 2018).

The bioinformatics analysis of relational networks between proteins that correlated with novel PE5 proteins was performed by ingenuity pathway analysis (IPA, Qiagen, Inc., Redwood City, CA, USA). The queried data sets that were generated for the PE5 proteins were significant as assessed by adjusted *p* value < 0.01 and included proteins with an expression correlation to a given PE5 protein across the samples in our study. Additionally, IPA provided overrepresented functional annotations and pathways within the identified subnetworks.

Protein family annotation (PFAM) of the PE2 proteins was detected using the DAVID bioinformatics database (Huang et al. 2009a, b). Spearman rank correlation test was performed to determine the correlation coefficient between PE2 proteins and protein members within the same family. The analysis was based on protein intensities that were quantitated considering unique peptides only. Correlations with *p* values < 0.05 were considered significant.

## Results and discussions

Well-characterised samples from 140 patients with stage 3 malignant melanoma (at the time of tissue collection) were investigated. A robust workflow (Fig. 2a) was implemented that combines an automated biobank platform, advanced high-throughput proteomics, and bioinformatics. Briefly, the tissue samples were collected from patients and stored with all clinical data in a quality-controlled biobank (Welinder et al. 2013). The samples were processed with modern and reproducible proteomic techniques. To obtain all possible information related to the identified proteins, the data generated was processed with a range of bioinformatics tools.

All novel peptides were mapped to Ensembl, Refseq, and UniProt with allowance for amino acid substitutions and gaps. The aim was to determine if variants of the same peptide were apparent in other proteins and could thus explain the mass spectra. More than 5000 possible variants were returned, but none passed the criteria, i.e., a tryptic peptide with a theoretical mass ± 10 ppm of the experimental mass.

All tumours are unique in morphology and underlying biological processes; however, some drivers are shared amongst melanomas. The high number of processed heterogeneous tumour tissues enabled the identification of 33 ‘missing proteins’ across the 140 samples (Table 1). All proteins were classified according to the PE category reported by neXtProt. Annotations were applied according to the HUPO guidelines, namely, ‘two or more distinct, uniquely mapping, non-nested peptide sequences per protein of length ≥ 9 amino acids’ (Omenn et al. 2017). After applying these guidelines, the number of missing proteins was reduced to nine (Table 1) and they can be divided into two groups (PE2 and PE5):

1. Proteins uniquely identified in this study within the context of metastatic cancer progression: Q9BSN7 (TMEM204), Q8N8Y5 (ZFP41), C9JJ37 (BTBD19), Q32M45 (ANO4) although previously supported only by transcript presence (**PE2**)
2. Proteins where the annotation was confirmed and explicitly linked the proteins to mechanisms of melanoma metastasis: Q58FG1 (HSP90AA4P), Q6ZTU2 (EP400P1), Q8IUI4 (Putative SNX29P2), Q58FF7 (HSP90AB3P), A0A0J9YWL9 (TEX13C) while previously marked as proteins of uncertain evidence and suspected to be pseudogenes (**PE5**)

**Table 1:** Total list of ‘missing proteins’ identified in this study. The first nine proteins were identified with at least 2 peptides with ≥ 9 amino acids

No.	Protein accession^a^	Gene symbol	Description	Chromosomal position	Peptide sequence	No. of total PSMs^b^	Coverage [%]^c^	No. of samples detected^d^	neXtProt PE level
1	C9JJ37	BTBD19	BTB/POZ domain-containing protein 19	1p34.1	VGAAVLERPVAEVAAPVVK	1	15	30	PE2
					QEVFAHR	1			
					LALLAPAELSALEEQNR	1			
2	Q32M45	ANO4	Anoctamin-4	12q23.3	ESSLINSDIIFVK	1	6	20	PE2
					LHAPWEVLGR	2			
					ETLPDLEENDCYTAPFSQQR	1			
					ISFPQWEK	1			
3	Q8N8Y5	ZFP41	Zinc finger protein 41 homologue	8q24.3	AFNCGSNLLK	5	20	70	PE2
					EEADVQK	3			
					TEPCLSPEDEEHVFDAFDASFK	2			
4	Q9BSN7	TMEM204	Transmembrane protein 204	16p13.3	GLDNDYVESPC	1	23	80	PE2
					SCWLVDR	1			
					GGPSPGAR	2			
					AGQVDAHDCEALGWGSEAAGFQESR	6			
5	A0A0J9YWL9	TEX13C	Putative testis-expressed protein 13C	Xq25	SRPWNEVEDR	1	4	10	PE5
					EMVPLGDSHSLK	1			
6	Q58FF7	HSP90AB3P	Putative heat shock protein HSP 90-beta-3	4q21-q25	SLTSDWEDHLAVK	3	36	40	PE5
					HLEINPDHPIMETLR	2			
7	Q58FG1	HSP90AA4P	Putative heat shock protein HSP 90-alpha A4	4q35.2	DLIMDNCEELIPEYLNFIR	9	19	58	PE5
					EDLELPEDEEEK	2			
8	Q8IUI4	SNX29P2	Putative protein SNX29P2	16p11.2	ESTQNVTLLK	7	30	60	PE5
					ESTQGVSSVFR	3			
9	Q6ZTU2-6	EP400P1	Isoform 5 of Putative EP400-like protein	12q24.33	QNDLDIEEEEEEHFEVINDEVK	2	25	80	PE5
					TSAAFPAQQQPLQVLSDGSTVQLPR	7			
10	Q5BKT4	ALG10	Dol-P-Glc:Glc(2)Man(9)GlcNAc(2)-PP-Dol alpha-1,2-glucosyltransferase	12p11.21	LNIPLPPTSR	15	23	110	PE2
11	Q7Z769	SLC35E3	Solute carrier family 35 member E3	12q15	AMTTPVIIAIQTFCYQK	6	10	140	PE2
					LSEQEGSR	18			
					LDIFAPK	18			
12	Q8TBE1	CNIH3	Protein cornichon homologue 3	1q42.13	SPIDQCNPVHAR	7	11	70	PE2
13	A1L157	TSPAN11	Tetraspanin-11	12p11.21	TLAENYGQPGATQITASVDR	1	17	10	PE2
					QVPDSCCK	1			
14	O14610	GNGT2	Guanine nucleotide-binding protein G(I)/G(S)/G(O) subunit gamma-T2	17q21	EYVEAQAGNDPFLK	8	39	70	PE2
15	O43374	RASA4	Ras GTPase-activating protein 4	7q22-q31.1	EAWMEPLQPTVR	2	38	20	PE2
16	P18825	ADRA2C	Alpha-2C adrenergic receptor	4p16.1	AGAEGGAGGADGQGAGPGAAESGALTASR	1	6	10	PE2
17	Q13304	GPR17	Uracil nucleotide/cysteinyl leukotriene receptor	2q21	TNESSLSAK	1	2	9	PE2
18	Q5VVM6	CCDC30	Coiled-coil domain-containing protein 30	1p34.2	QHNSLLQEENIK	4	3	40	PE2
					ELELEVLK	1			
19	Q7Z602	GPR141	Probable G protein coupled receptor 141	7p14.1	YGIHEEYNEEHCFK	1	7	10	PE2
20	Q86X67	NUDT13	Nucleoside diphosphate-linked moiety X motif 13	10q22.3	DASLLSTAQALLR	3	6	69	PE2
					HSLLELER	4			
21	Q8IY85	EFCAB13	EF-hand calcium-binding domain-containing protein 13	17q21.32	EILEEVTK	5	3	49	PE2
					ILQSDFVSEDNMVNIK	1			
22	Q9UPC5	GPR34	Probable G protein coupled receptor 34	Xp11.4	IMYHINQNK	3	9	90	PE2
					FPNSGK	2			
					YATTAR	4			
					IMCQLLFR	2			
					FQGEPSR	1			
23	Q9Y5I0	PCDHA13	Protocadherin alpha-13	5q31	VTVLENAFNGTLVIK	14	6	80	PE2
24	A0A0A0MT36	IGKV6D-21	Immunoglobulin kappa variable 6D-21	2p11.2	YASQSISGVPSR	8	16	70	PE3
25	A0A075B6S6	IGKV2D-30	Immunoglobulin kappa variable 2D-30	2p11.2	VSNWDSGVPDR	3	44	30	PE3
26	Q9BZK3	NACA4P	Putative nascent polypeptide-associated complex subunit alpha-like protein	8q22.3	IEDLSQEAQLAAAEK	27	13	140	PE5
27	Q58FF3	HSP90B2P	Putative endoplasmin-like protein	15q26.3	EFEPLPNWVK	20	24	130	PE5
28	Q96L14	CEP170P1	Cep170-like protein	4q26	EINDVAGEIDSVTSSGTAPSTTLVDR	9	58	90	PE5
29	Q9BYX7	POTEKP	Putative beta-actin-like protein 3	2q21.1	LCYVALDSEQEMAMAASSSSVEK	1	29	70	PE5
					RGMLTLK	12			
30	Q8NF67	ANKRD20A12P	Putative ankyrin repeat domain-containing protein 20A12 pseudogene	1q12	LEEIHLQEQAQYK	1	11	10	PE5
31	A2A3N6	PIPSL	Putative PIP5K1A and PSMD4-like protein	10q23.33	SNPENNVGLITLDNDCEVLTTLTPDTGR	1	24	10	PE5
32	Q8IX06	REXO1L1P	Putative exonuclease GOR	8q21.2	LQEFLLTQDQLK	1	4	10	PE5
33	P0CG22	DHRS4L1	Putative dehydrogenase/reductase SDR family member 4-like 1	14q11.2	LGEPEDSLGIVSFLCSEDASYLTGETVMVGGGTPSR	2	29	7	PE5

^a^Protein accession number in UniProt database

^b^Total number of peptide-spectrum matches for the particular peptide sequence

^c^Percentage of the protein sequence identified with unique and non-unique peptides

^d^Number of melanoma tumour samples for which the protein was identified

The remaining 24 proteins were identified in up to 140 melanoma metastases (Table 1). As most of the missing proteins are possibly low-abundance proteins (Wei et al. 2016), these results can be considered as further evidence to support the existence of the proteins in the tumours.

Expression correlation is known to be an indicator of functional association between genes or proteins (Pita-Juárez et al. 2018). Four individual Spearman correlation tests were performed to determine if there are any possible functional associations between the four PE2 proteins and well-known proteins with similarities in function, structure, or sequence. Using the protein intensities obtained from the MS data, for each novel protein, the correlation was assessed against proteins that have the same PFAM structural domains.

Two of the four proteins annotated as PE2 (Q8N8Y5/ZFP41 and C9JJ37/BTBD19) were significantly correlated with proteins possessing the C2H2 zinc finger domain (PF00096) and BTB/POZ domain (PF00651), respectively. Each protein was individually associated with one different protein family as shown in Fig. 2B. The Q32945/ANO4 protein was not significantly correlated with any proteins of the same family (anoctamin, calcium-activated chloride channel, PF04547); and the Q9BSN7/TMEM204 protein does not have close human homologues in existing protein databases. FFAS analysis of remote sequence similarities, however, showed that TMEM204 is significantly similar to claudin-like transporters that have known roles in tight junction and in cancer.

The zinc finger domain proteins have often been related to cancer progression, including several cancer forms, such as breast cancer, gastric cancer, and melanoma (Cassandri et al. 2017; Lim 2014a). To date, however, specifically, the ZFP41 gene has never been differentially detected in any melanoma study. As this constitutes the first evidence at the protein level, future studies are necessary to relate the expression of the protein with the progression of melanoma.

Conversely, the BTB/POZ domain-containing proteins are known to be involved in several types of human cancer (Nakayama et al. 2006). The BTBD19 gene has been differentially expressed in melanoma studies (Expression Atlas codes (https://www.ebi.ac.uk/arrayexpress/experiment): E-MTAB-6214, E-MTAB-7143). As this is the first time that C9JJ37/BTBD19 has been observed on protein level, future studies should be performed to confirm the specific role of this protein in melanoma. Of note, both domains (zinc finger and BTB/POZ) are structural sections of the proteins termed ‘ZBTB’, an emerging family of transcription factors with active roles in oncogenesis (Lee and Maeda 2012; Lim 2014b).

In addition, expression data analysis of the PE2 proteins (genes: BTBD19, ANO4, ZFP41, and TMEM204) revealed that all have been previously observed in skin tissues, melanoma cell lines, or melanoma tissues (Fig. 2C). This evidence was provided by The Human Protein Atlas (Uhlén et al. 2016; Thul and Lindskog 2018). Taken together, the results are highly supportive of the presence of such proteins in stage 3 melanoma.

Seven of the nine proteins were quantitated in more than 30 samples and all nine in more than 10 samples. The identification frequencies of the nine PE2 and PE5 proteins during the whole analysis are shown in Fig. 2D.

**Fig. 3:**
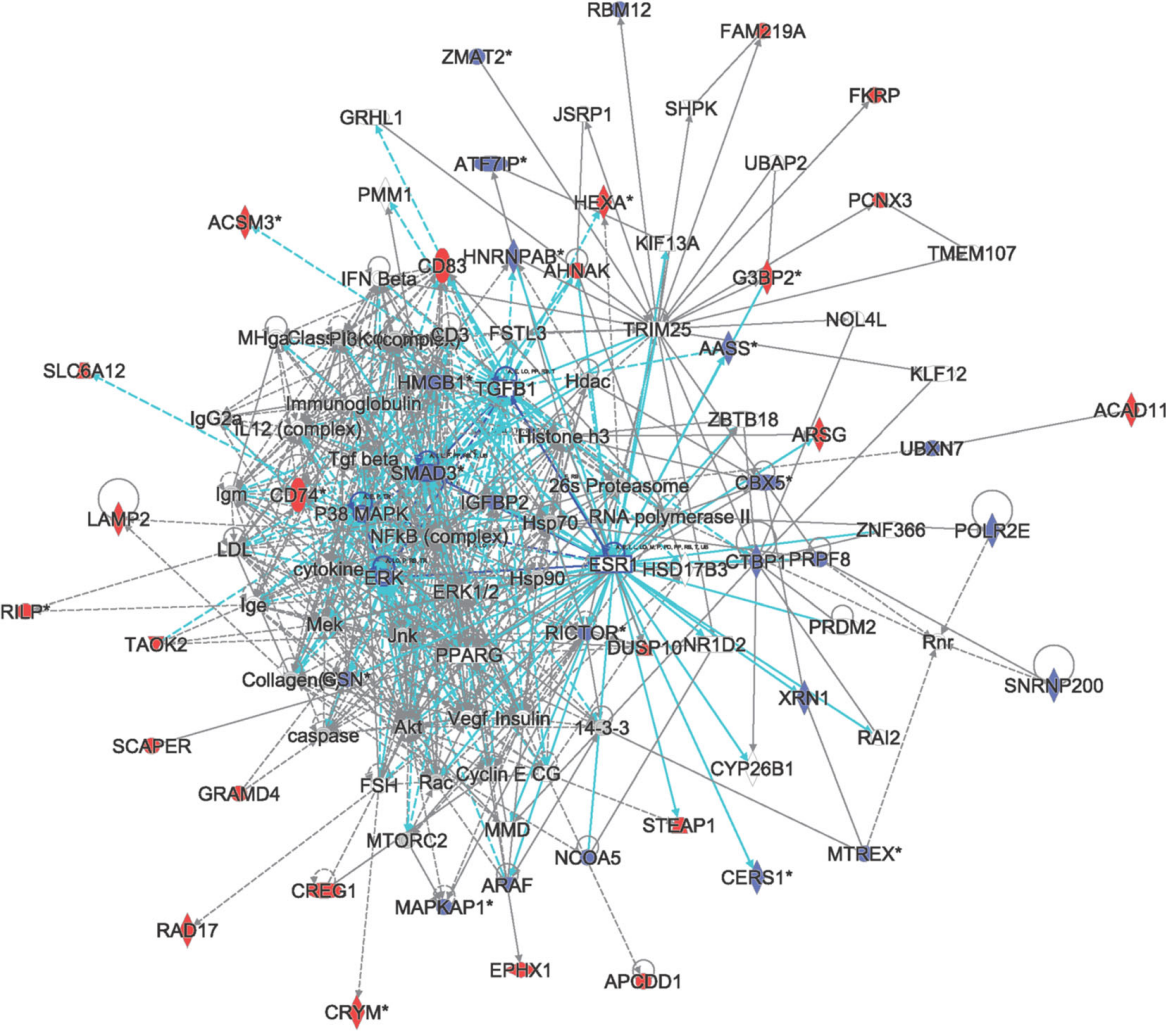
Functional relationship network for proteins correlated to TEX13C. Ingenuity pathway analysis (IPA) for the proteins significantly correlated to TEX13C expression in the melanoma samples. Three top protein-protein functional relationship subnetworks merged. Red, proteins with expression positively correlated to TEX13C. Blue, proteins negatively correlated to TEX13C. Solid lines, direct functional relationships. Dashed lines, indirect relationships

Five of the nine novel proteins were annotated previously as the ‘suspect’ PE5 proteins (Table 1). Proteins annotated as PE5 typically have little to no information in the literature. Therefore, sets of proteins with expression patterns across the melanoma samples that correlated with the PE5 proteins identified in this study were queried. IPA provided functional relational subnetworks enriched in the correlated proteins. For TEX13C, IPA analysis of the correlated proteins resulted in a relational network that centred on hubs known for their involvement in cancer, such as the oestrogen receptor ESR1, SMAD3 (Tang et al. 2017), TGFB1, and ERK/MAPK kinases (Fig. 3). The proteins correlated with TEX13C are involved in cell-to-cell signalling and interaction, cellular growth and proliferation, and RNA post-transcriptional modification. For the proteins that significantly correlated with TEX13C expression, IPA generated the top three protein-protein functional relational subnetworks. TEX13C (LOC100129520) is a member of the TEX13 family that is comprised of two other members, TEX13A and TEX13B. The latter two proteins have been characterised to some extent. TEX13A is an RNA-binding protein (Nguyen et al. 2011) and the mouse homologue is a male germ cell–specific nuclear protein that may be involved in transcriptional repression (Kwon et al. 2016). This protein possesses an uncharacterised structural domain termed TEX13 and a zinc finger domain zf-RanBP (PFAM:PF00641).

Two putative HSP90 heat shock proteins, HSP90AA4P and HSP90AB3P, are close homologues of the HSP90 chaperones with well-known roles in cancer and well-established as cancer drug targets (Mbofung et al. 2017). In this study, we could establish protein-protein correlations, where 527 and 242 proteins were found to be significantly correlated with HSP90AB3P and HSP90AA4P, respectively. IPA analysis of these protein data sets yielded RNA post-transcriptional modification as the top, overrepresented functional annotation. Other overrepresented functional annotations included molecular transport and RNA trafficking for HSP90AB3P, and protein synthesis and cell morphology for HSP90AA4P.

The EP400P1 protein is a homologue of the E1A-binding chromatin remodeller EP400, albeit containing only the EP400_N domain with unknown function (Elsesser et al. 2019) and lacking a catalytic DEAD nuclease domain. Such an arrangement may indicate a regulatory function that is related to the longer homologue, EP400. A large number of proteins correlated with the expression of EP400P1 and the top functional annotations of the group were a cellular compromise, molecular transport, and cellular assembly and organisation.

The SNX29P2 protein is a homologue of sorting nexins involved in endosomal retromer complex function (Gallon and Cullen 2015), although the protein lacks important functional domains (the RUN domain that is probably involved in Ras-like GTPase signalling pathways and the phosphatidylinositol-3-phosphate-binding PX_RUN domain). As such, SNX29P2 can be hypothesised as a modulator of the full-length homologue, sorting nexin-29. A very large set of proteins was observed to correlate with the expression of SNX29P2 and IPA revealed that cellular development, cellular growth and proliferation, and cell death and survival were the most common annotations amongst these proteins. Overall, the sets of proteins that had expression levels in the melanoma samples that correlated with the five novel PE5 proteins are indicative of cancer-related functions.

In conclusion, new protein evidence for nine ‘missing proteins’ is reported. These were expressed in lymph node metastases of malignant melanoma. The proteins were clearly identified across a large-scale analysis of clinical samples from melanoma patients. Furthermore, associations with cancer-related functions were obtained and discussed for all the reported proteins.
